# Co-occurrence of CML Blast Crisis and Severe COVID 19 Infection: A Case Report

**DOI:** 10.7759/cureus.26865

**Published:** 2022-07-14

**Authors:** Misbahuddin Khaja, Vibha Hayagreev, Asim Haider, Diana Ronderos, Ayesha Siddiqa, Valentina Moirangthem

**Affiliations:** 1 Internal Medicine/Pulmonary Critical Care, BronxCare Health System, New York, USA; 2 Internal Medicine, BronxCare Health System, New York, USA; 3 Hematology and Oncology, BronxCare Health System, New York, USA

**Keywords:** imatinib therapy, invasive mechanical ventilation, corona virus disease 2019, chronic myeloid leukemia (cml), blast crisis

## Abstract

Coronavirus disease 2019 (COVID-19) is caused by severe acute respiratory syndrome coronavirus 2 (SARS-CoV-2) affecting multiple organ systems. It can cause severe cytokine storms leading to intensive care unit admission requiring mechanical ventilation. However, there have been few studies establishing the outcomes of chronic myeloid leukemia (CML) patients on tyrosine kinase inhibitors who are infected with COVID-19. We present a 69-year-old male with a history of CML on imatinib therapy with COVID-19 who developed acute respiratory distress syndrome needing mechanical ventilatory support, shock requiring vasopressors, and worse outcome secondary to blast crisis.

## Introduction

Chronic myeloid leukemia (CML) is a myeloproliferative neoplasm that comprises mainly a chronic phase, very few transform as blast phase or accelerated phase. Malignancy can increase susceptibility to infections as it is an immunosuppressive state [1]. Several acute respiratory symptoms were reported in December 2019 that led to the detection of the coronavirus outbreak. Later this new strain was confirmed and identified in January 2020 as severe acute respiratory syndrome coronavirus 2 (SARS-CoV-2) [2].

Few studies found that patients with hematologic malignancies who are infected with COVID-19 infection had an increased rate of complications and mortality when compared to the rest of the population [3]. The first study reported in February 2020 showed that SARS-CoV-2 infection rates in patients with cancer were 0.7% compared to non-cancer subjects, which was 0.37%, followed by multiple studies that were cohorted showing similar results [4].

Studies showed that patients diagnosed with hematological malignancy and COVID 19 infection had a mortality rate estimated to be 34%, however, the percentage estimated may have been biased by a high number of patients hospitalized in published studies [5].

## Case presentation

This is a case of a 69-year-old male with a past medical history of CML, bronchial asthma, chronic lower back pain, and hypertension. He was started on imatinib by a hematologist as an outpatient for his CML. He was referred to the emergency room complaining of abdomen pain after starting Imatinib. In the emergency room, vitals were significant for tachycardia of 124/minute, blood pressure of 118/59 mmHg, afebrile, and saturating 94% on room air. Laboratory findings were significant for leukocytosis with a white blood cell count of 66.1 k/ul, anemia with hemoglobin of 8.4 g/dl, and thrombocytopenia with a platelet count of 34 k/ul, acute kidney injury, hyperkalemia, and hyperuricemia (Table 1).

**Table 1:** Detailed laboratory results BUN: Blood urea nitrogen, CRP: C-reactive protein, LDH: Lactate dehydrogenase

Day	1	2	3	4	5	6	7	8
WBC (4.8-10.8 K/ul)	66.1	73.7	84	52.3	32.7	21.8	18.1	16.8
Hemoglobin (12.0-16.0 g/dl)	8.4	7.3	7.1	6.9	7.6	7.5	6.9	7
Platelets (150-400 k/ul)	34	27	27	43	39	24	17	28
BUN (8.0-26.0 mg/dl)	41	35	52	81	110	102	130	107
Creatinine (0.5-1.5 mg/dl)	1.9	1.5	2.4	3.4	5.1	5.3	7.1	6.9
Potassium (3.5-5.0 mEq/L)	5.5	5.8	5.1	6.7	6.9	6.6	6.8	7.4
Uric Acid (2.5-8.0 mg/dl)	8.3	7.2			19.9		20.5	
D-Dimer (0-230 ng/mL)	2752						3403	
CRP (< 5.0 mg/L)	147.92							
LDH (110-210 uni/L)	1780							

**Figure 1:**
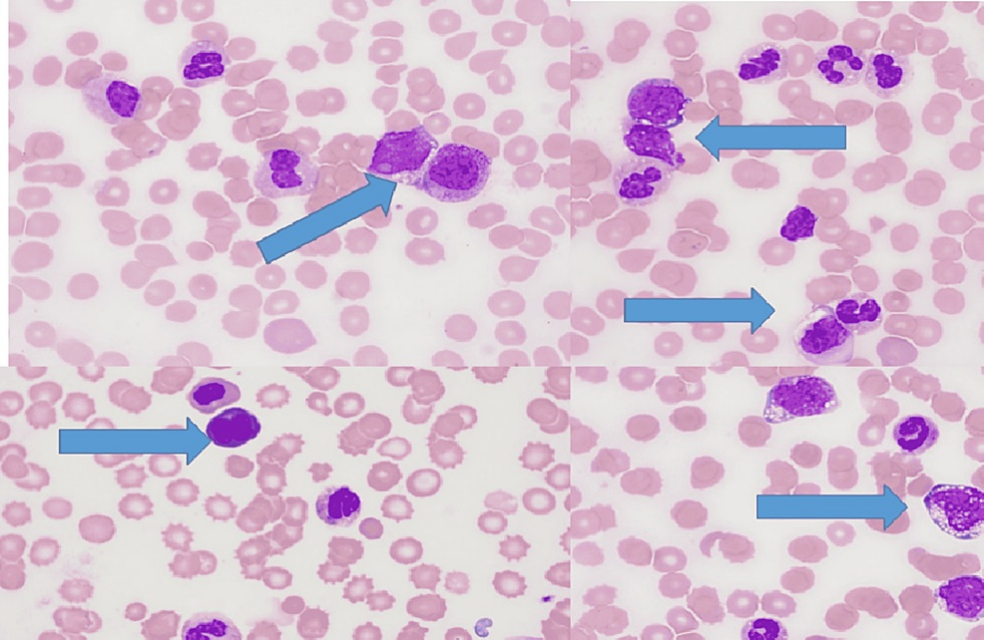
Peripheral blood smear blue arrow showing basophilia, granulocytosis with neutrophils, and immature granulocytes

The patient’s peripheral smear showed about 8% blast cells suggestive of accelerated blast crisis (Figure 1).

**Figure 2:**
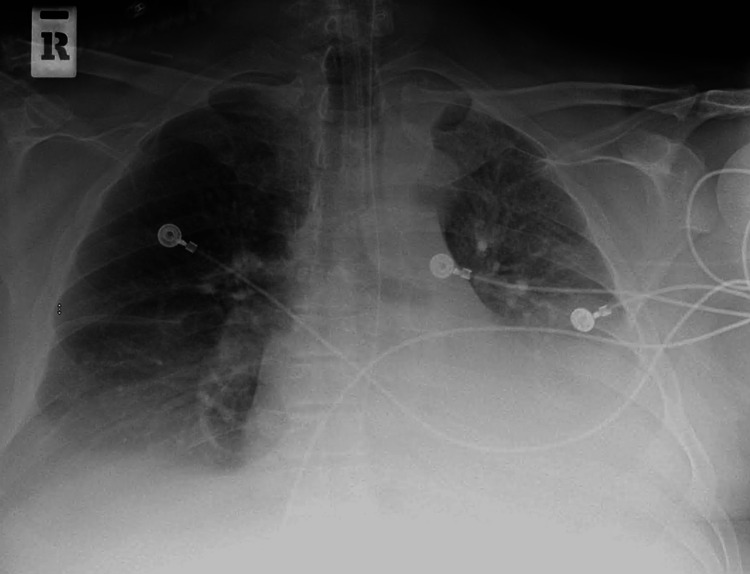
X-ray chest showing bilateral (left greater than right) interstitial and bibasilar infiltrates with pleural effusion

A CT scan of the abdomen was negative for any acute changes. The patient was noted to have a positive polymerase chain reaction (PCR) assay for SARS-CoV-2 in the nasopharyngeal swab. He was admitted to the intensive care unit. His respiratory status worsened requiring mechanical ventilation. Chest X-ray showed bilateral interstitial prominence and bilateral lower lobe infiltrates (Figure 2). 

The patient was treated with broad-spectrum antibiotics (vancomycin and piperacillin/tazobactam), and a septic workup which was negative for any bacterial infection. The worsening renal function, hyperuricemia, and hyperkalemia led to the diagnosis of tumor lysis syndrome. The nephrology service was consulted and he was started on hemodialysis. Hematology was consulted for worsening leukocytosis, thrombocytopenia, and peripheral smear showing blast cells, so the patient was restarted on Imatinib. Peripheral blood flow cytometry revealed an increased blast population comprising 30% of the total analyzed cells.

The patient went into acute respiratory distress syndrome, shock requiring multiple pressors. He received steroids (dexamethasone 6mg intravenous twice daily) and broad-spectrum antibiotics. Unfortunately, he was not a candidate for remedesivir secondary to worsening renal function. He was given one dose of tocilizumab with no improvement in his inflammatory markers. Due to his worsening medical condition, he did not survive.

## Discussion

Chronic myeloid leukemia is a myeloproliferative neoplasm in which up to 90% are present in the chronic phase, the rest in the blast phase or accelerated phase. The disease is characterized by reciprocal chromosomal translocation resulting in the BCR-ABL 1 fusion gene. These phases of CML are differentiated by myeloblast count, chromosomal abnormalities, basophil percentage, and clinical pathologic features. Some chronic phases can be transformed into accelerated phases while treated with a tyrosine kinase inhibitor. The exact mechanism of how the transformation of the chronic phase to the blast crises phase is still undetermined [6]. The CML blast crisis (CML-BC) comprises 30% of lymphoid blast crises and 70% of myeloid blast crises [7].

As per European Leukemia Net (ELN) criteria, CML-BC has more than 30% of the blast, also ≥30% as per MD Anderson Cancer Center and the International Bone Marrow Transplant Registry criteria whereas World Health Organization criteria have more than 20% of the blast. The molecular or cytogenetic abnormalities also play a role in the classification and staging of CML. The patient in CML-BC can present with fever, shortness of breath, abdomen pain, infections, decreased appetite, weight loss, bleeding, bone pain, and splenomegaly. They also present with anemia, high white blood cell count, and very low or elevated platelet count. Blast cells spread to other tissues and organs from bone marrow, blood, and CML cells with new chromosomal abnormalities [8].

The risk factors for blast crises include the history of CML, exposure to alkylating chemotherapy, and exposure to ionizing radiation. The investigation consists of a peripheral blood smear, bone marrow aspiration, biopsy, fluorescence in situ hybridization, and quantitative reverse-transcriptase polymerase chain reaction. Although the timing of T-cell blast crisis from the CML chronic phase is not well understood, it can range between three to 48 months but can also happen in one month after stopping imatinib therapy like our patient [9].

There are prognostic factors that predict a patient's recovery and treatment. Patients who have blast phase or accelerated phase have a poor prognosis. Age is also considered a prognostic factor. The older the patient poorer the prognosis. A higher blast count in the blood has a poor prognosis. An enlarged spleen has a poor prognosis. Very high, very low platelets are also considered a less favorable outcome. An increase in eosinophils or basophils in the blood is considered to have a poor prognosis [10].

Beta coronavirus family SARS-COV-2 enters the lung by binding its spike (S) protein on its surface to angiotensin-converting enzyme receptor 2. Its transmission is by direct contact of respiratory droplets from an infected patient to others. The inflammatory storm is caused by overproduction of interleukine six and tissue factors contributing to a hypercoagulable state. Unfortunately, there are not enough studies to conclude the true impact of this COVID-19 infection on CML [11].

The prevalence of COVID-19 infection was nine times higher in CML patients as per Li et al.'s study. In their research, most CML patients diagnosed with COVID-19 infection were in the blast or accelerated phase, on treatment with imatinib [12]. The blast phase has a poor prognosis because of bleeding and the risk of developing superimposed infections that are challenging to manage due to resistance to tyrosine kinase inhibitors. In addition, tyrosine kinase inhibitors can be intolerable in COVID-19 patients due to their risk of side effects like fluid retention, pulmonary toxicity, myelosuppression, and thrombosis [13,14].

The COVID-19 infection can be higher in cancer patients. In COVID-19 patients, proinflammatory cytokines lead to lung injury. In addition, the smoking history in cancer patients is associated with the expression of angiotensin-converting enzyme 2, which is the binding receptor for SARS coronavirus. This could be one of contributing factors in a cancer patient acquiring COVID-19 infection [15]. A study by He et al. showed that hematologic malignancies have a severe clinical course by superimposed infection or progression of malignancy itself and do not risk acquiring COVID-19 infection [16].

Treatment of blast crisis of CML aims to revert to chronic phase and finally to get allogeneic hematopoietic cell transplantation once stable. The myeloid blast crisis is treated with a tyrosine kinase inhibitor. If a blast crisis develops while the patient is on a tyrosine kinase inhibitor, a more potent tyrosine kinase inhibitor is given with acute myeloid leukemia (AML) type induction chemotherapy which includes a seven-day continuous infusion of cytarabine plus an anthracycline for three days known as 7+3 therapy, for the remission [17]. Tyrosine kinase inhibitor used is imatinib in treating blast crisis. The second-generation tyrosine kinase inhibitors like nilotinib and dasatinib are used if there is disease progression, or the patient is already receiving imatinib. Molecular or cytogenetic abnormalities or both are used to check on response to treatment or failure [18].

There are few case reports where tocilizumab was used in a patient with hematologic malignancy co-occurrence of SARS-CoV-2 infection. However, successful use of tocilizumab in a patient with multiple myeloma with SARS-CoV-2 infection was shown in a case report by Zhang et al. [19]. Another case report of a 52-year Indian male with poorly controlled CML not responding to imatinib by Ranger et al. had COVID-19 infection which responded to tocilizumab; his inflammatory marker, oxygen requirement decreased and helped in cytokine release syndrome [20]. 

Prognosis is abysmal despite treatment of blast crisis in CML, as this phase is resistant to most treatments. There is a significant relapse even after treatment and should be referred for allogeneic hematopoietic cell transplantation.

Our patient had a history of CML on imatinib, developed blast crisis phase, and was critically ill secondary to COVID-19 whose cytokine storm did not respond to tocilizumab and steroids.

## Conclusions

The blast crisis phase in CML is the final stage, with high mortality. Multiple immature white blood cells in blood, organs, bone marrow, and tissues make it fatal without treatment. Chronic myeloid leukemia patients who develop COVID-19 infection are a significant challenge for a physician to treat during a pandemic. Though the antiviral activity of tyrosine kinase inhibitors prevents viral entry into cells, the outcome is poor once CML patients are in the blast crisis phase. There is a need for more studies to define and better understand the management of a patient in the blast crisis phase of CML infected with COVID-19.
